# 

**Published:** 1884-05

**Authors:** 


### CONTENTS.

COMMUNICATIONS.

The Debts We Owe........ 215
Address to the Graduates........ 224
Operative Dentistry........ 232
Cause and Treatment of Facial Neuralgia........ 243
A New Observation in Dentinal Structure........ 245
Extracts........ 248

PROCEEDINGS.

Proceedings of Societies........ 252

SELECTIONS.

Patients will Lie........ 255
Why is Chromic Acid such a Valuable Caustic?........ 255
How to Keep the Hypodermic Syringe in Order........ 255
The M. D.'s........ 256
Errors in Diagnosis........ 256
SALMAGUNDI........ 257
EDUCATIONAL........ 262
EDITORIAL........ 266

